# Pharmaceutical/Clinical Strategies in the Treatment of Acute Promyelocytic Leukemia: All-Trans Retinoic Acid Encapsulation by Spray-Drying Technology as an Innovative Approach–Comprehensive Overview

**DOI:** 10.3390/ph16020180

**Published:** 2023-01-24

**Authors:** Antónia Gonçalves, Fernando Rocha, Berta N. Estevinho

**Affiliations:** 1 LEPABE—Laboratory for Process Engineering, Environment, Biotechnology and Energy, Department of Chemical Engineering, Faculty of Engineering, University of Porto, Rua Dr. Roberto Frias, 4200-465 Porto, Portugal; 2 ALiCE—Associate Laboratory in Chemical Engineering, Faculty of Engineering, University of Porto, Rua Dr. Roberto Frias, 4200-465 Porto, Portugal

**Keywords:** acute promyelocytic leukemia, all-trans retinoic acid, carrier-based delivery systems, encapsulation technology, promyelocytic gene, retinoic acid receptor α

## Abstract

Acute promyelocytic leukemia (APL) is phenotypically characterized by the accumulation of dysplastic promyelocytes, resulting from a cytogenetic condition due to the balanced chromosomal translocation t(15;17)(q22;q21). Current first-line treatment of APL includes all-trans retinoic acid (all-trans RA), with or without arsenic trioxide, combined with chemotherapy, and a chemotherapy-free approach wherein arsenic trioxide is used alone or in combination with all-trans RA. The usage of all-trans RA revolutionized the treatment of APL, with survival rates of 80 to 90% being achieved. The mechanism of action of all-trans RA is based on regulation of gene transcription, promoting the differentiation of leukemic promyelocytes. Encapsulation technology has been explored as an innovative strategy to overcome the major drawbacks related to the all-trans RA oral administration in the APL treatment. The most recently published works on this subject highlight the development and optimization of carrier-based delivery systems based in microparticle formulations obtained by spray-drying to be used in the treatment of APL. The ultimate goal is to obtain a controlled delivery system for RA oral administration capable of providing a slow release of this bioactive compound in the intestinal lumen.

## 1. Introduction

Acute promyelocytic leukemia (APL) was first described by the hematologist Hillestad in 1957. Three patients evidenced a very rapid decline and death (with only a few weeks duration), which occurred as a result of the accumulation of dysplastic (abnormal) promyelocytes and severe hemorrhagic conditions due to fibrinolysis and thrombocytopenia. These key elements enabled APL to be recognized as a subtype of acute myeloid leukemia (AML) [1,2,3]. APL was then fully characterized by Bernard in 1959 when twenty patients evidenced bone marrow infiltration by abnormal promyelocytes, with observation of acute hemorrhagic episodes and the rapid evolution of disease due to hyperfibrinolysis and/or disseminated intravascular coagulation [1,3].

APL is known as M3 by the French–American–British classification and accounts for 5 to 10% of AML in adults [4,5]. A recent study performed by Dinmohamed and Visser [6] updates on the incidence of APL across Europe. The authors pointed out the scarcity of studies performed over the past few decades regarding this topic in Central and South America. The incidence of APL was variable across Europe, with the highest incidence in Spain.

APL was reported as a rapidly fatal disease until the late 1980s [7]. Currently, it is considered the most curable subtype of AML with survival rates of 80% to 90%, with the combination of the all-trans-RA and arsenic trioxide (non-chemotherapy regimen) [8,9]. Eventually, the combination of these compounds and chemotherapy was also considered in the treatment of APL [7,9,10,11].

This review provides an overall discussion about the identification, diagnosis and treatment course of APL. Furthermore, the mechanism of action of all-trans retinoic acid (all-trans RA), based on the genetic hallmark of APL, is described for the treatment of this disease. The last section focuses on the most recent strategies to overcome the main challenges related to the current clinical usage of all-trans RA for the treatment of APL and includes the encapsulation of all-trans RA into carrier-based delivery systems.

## 2. Identification and Diagnosis of APL

**Figure 1:**
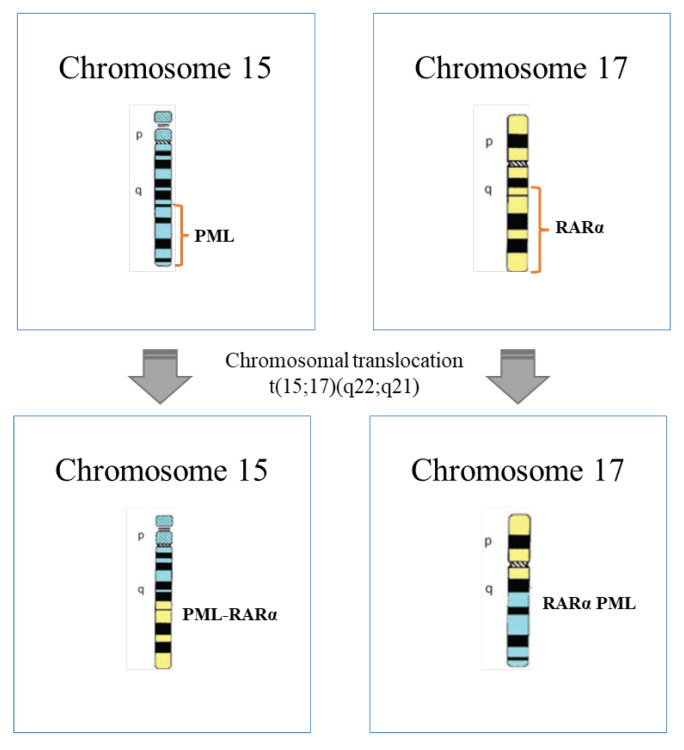
Schematic representation of chromosomal translocation t(15;17)(q22;q21) that occurs at APL (adapted from [26]).

APL has its origins in the PML/RARA fusion protein due to the balanced translocation between the promyelocytic (PML) and the retinoic acid receptor α (RARα) genes located on chromosome 15 and 17, respectively (Figure 1) [4,12,13]. The obtained oncoprotein blocks the differentiation of leukemic promyelocytes, inducing leukemia [14]. This chromosomal translocation represents 95 to 98% of APL cases and was recognized by Rowley in 1977 as a karyotypic change characteristic of this disease [15,16].

APL diagnosis can be first performed by the morphological characterization of APL. The abnormal promyelocytes can present a typical hyper-granular form, with a bilobed or reniform nuclear membrane, a densely granulated cytoplasm and Auer rods or Faggot cells (cells with bundles of Auer rods) [17,18]. In turn, the hypo-granular (microgranular) form of abnormal promyelocytes—observed with less frequency—presents bilobed nuclei, several sub microscopic granules and only a few cells with multiple Auer rods. The hyper-granular form often presents with a leukopenia, while the hypo-granular form commonly presents with a leukopenia [19,20,21,22]. The immunophenotype of blast cells in APL can be also evaluated for the diagnosis [23,24]. A more accurate approach can be performed at genetic level considering the cytogenetic characterization of APL, fluorescence in situ hybridization (FISH), reverse transcription polymerase chain reaction (RT-PCR) and immunofluorescence with anti-PML antibodies [19,25,26,27].

## 3. Treatment of APL

The pre-therapeutic era of APL occurred between 1940 and 1973, when the promyelocytic cell was first fully recognized. At that time, most of the APL patients died within four weeks, with only 6% to 14% of cases achieving remission [28].

The first approach proposed for the treatment of APL was based on the administration of anthracyclines to inhibit the proliferation of malignant cells [3,19]. Herein, patients treated with daunorubicin evidenced an increased complete remission rate from 13% to 55% [29]. Treatments based on anthracycline were later proven to be effective and showed to be dose-dependent, with improvement of remission rates with higher doses of daunorubicin [30]. Broadly, complete remission rates between 55% and 88% were observed, with 35% to 45% of patients entering a prolonged remission [19]. In fact, introduction of chemotherapy for APL treatment markedly changed the course of this disease [31]. The chemotherapy era lasted from 1973 to 1988.

The knowledge of the differentiation, proliferation and apoptosis of leukemic cells provided a new perspective for the APL treatment, with identification of some compounds capable of triggering the differentiation of these cells [3]. Accordingly, in the early 1980s, retinoic acid (RA) was reported to be capable of inducing morphologic and functional maturation of HL 60 cells, with identification of the specific response of APL specimens to RA [32,33]. In 1988, the efficacy of all-trans RA in the treatment of APL was first recognized, with achievement of complete remission with differentiation of promyelocytes in 23 of 24 patients studied at a dose from 45 to 100 mg·m^−2^·d^−1^ [34]. This was the beginning of the modern era in APL treatment.

The treatment of APL only with all-trans RA enables a complete remission rate of around 85% [3]. However, due to the continuous treatment with this drug, the patients become resistance to all-trans RA. Moreover, the amount of all-trans RA in the plasma greatly decreases, with relapse occurring within 3 to 6 months. At last, an increase in the amount of white blood cells with fatal RA syndrome (the symptoms of this syndrome include fever, acute renal failure, pleuro-pericardial effusion, hypotension, dyspnea, interstitial pulmonary infiltrates, peripheral edema and weight gain higher than 5 kg [25,35]) may be observed due to all-trans RA administration [3]. In line with these topics, the combination of all-trans RA and chemotherapy was proposed for the treatment of APL [25]. The studies indicated rates of cure higher than 80%. The Gruppo Italiano per le Malattie EMatologiche dell’Adulto (GIMEMA) and the Programa Espanol para el Tratamiento de las Hemopatias Malignas del Adulto (PETHEMA) used simultaneously all-trans RA and idarubicin (an anthracycline) as chemotherapy to induce remission, followed by three cycles of consolidation and maintenance therapy [36,37,38]. This approach—AIDA—has become one of the most widely used protocols. In 2000, the original protocols were updated with the use of less intense chemotherapy schedules for patients of low and intermediate risk [36,39,40]. The combination of all-trans RA and chemotherapy deals with severe hematologic toxicity, occurring in 2% to 3% of cases deaths in remission together with secondary myeloid neoplasms [25].

A study performed in 2004 with APL patients for induction therapy compared the treatment with arsenic trioxide or all-trans RA (each one individually used) or the combination of these two drugs [41]. The obtained results evidenced similar percentages of complete remission—between 90% and 95.2%—among all the approaches tested. However, the median time required to achieve complete remission was shorter when the combination of arsenic trioxide and all-trans RA was considered (25.5 days, instead of 40.5 or 31 days when all-trans RA and arsenic trioxide were individually used, respectively). All the patients were then (after induction) subjected to chemotherapy and the only one treated with the combination of arsenic trioxide and all-trans RA did not relapse. The estimated 5-year event-free survival and overall survival for patients treated with this combination of drugs were 89.2% and 91.7%, respectively. These results promoted investigations regarding the importance of arsenic trioxide in the consolidation of therapy used so far, with registration of significantly superior event-free survival, 3-year overall survival and 3-year disease free survival [42]. The induction therapy also showed the benefit of combining anthracycline chemotherapy, arsenic trioxide and all-trans RA followed by two consolidation cycles with the combination of arsenic trioxide and all-trans RA without chemotherapy, causing an improvement of 2-year freedom from relapse (97.5%), failure-free survival (88%) and overall survival (93%) [43].

A chemotherapy-free approach was investigated when arsenic trioxide was used for induction and consolidation therapy, with observation of significantly better outcomes for patients with low-risk disease [44,45]. The same approach was studied using the combination of arsenic trioxide and all-trans RA for induction and consolidation therapy [46]. Herein, complete remission rates and an estimated 3-year overall survival were 92% and 96%, respectively. Moreover, significantly better results were achieved for patients with low-risk disease. In another study, the Italian cooperative group GIMEMA worked in collaboration with the German AMLSG and SAL cooperative groups to perform randomized studies to compare the combination of all-trans RA and chemotherapy against the chemotherapy-free approach [47]. Herein, the combination of arsenic trioxide and all-trans RA was administrated for the induction therapy, followed by four consolidation cycles with intermittent arsenic trioxide and all-trans RA. The obtained results regarding event-free survival and overall survival were better when compared to the standard AIDA, in addition to being associated with significantly less myelosuppression and infections. However, the combination of arsenic trioxide and all-trans RA enabled an increase in the liver enzymes and the observation of QTc prolongation, which can be controlled and reversible with temporary drug discontinuation. In line with this study, the NCCN guidelines were updated, with identification of the combination of arsenic trioxide and all-trans RA as the most auspicious option for the front-line therapy of APL patients with low and intermediate risk. Comparing the combination of these drugs with the combination of all-trans RA and chemotherapy, the first one showed superior results of event-free survival and overall survival and a significantly lower incidence of relapse [48]. For the APL patients with a high risk, it was of major importance to compare the combination of arsenic trioxide, all-trans RA and minimal chemotherapy against AIDA [25]. Some of the most recently updated recommendations on the management of acute promyelocytic leukemia were published in 2019 by an expert panel of the European LeukemiaNet [49].

## 4. Mechanisms of Action of All-Trans RA

The cellular mechanism of all-trans RA in the treatment of APL is based on triggering the differentiation of immature neoplastic cells into mature granulocytes [50]. Briefly, it has been considered that all-trans RA acts on two stages of myeloid cell development—promyelocytes and earlier neoplastic progenitor cells [51]—wherein a differentiation step induced by this bioactive compound is followed by the apoptosis of the maturing cells originating from the leukemic clone [50]. A two-step model in the maturation process explains the differentiation of APL cells by all-trans RA, which includes the proliferation and simultaneously the cells become competent (RA-dependent step); and cellular terminal maturation (cyclic adenosine monophosphate (cAMP)-dependent step) [50]. An activation mechanism involving the nuclear all-trans RA, cAMP and the protein kinase A (PKA) pathway is proposed here [52].

Retinoids regulate gene transcription mediated through their binding to RA receptors (RARs) and retinoid X receptors (RXRs) [53,54]. In particular, all-trans RA activates RARs [55]. In the presence of all-trans RA, their binding to the heterodimer RAR/RXR (the biological active form of RARα) activates a conformation change and the formation of a ligand receptor complex. The obtained structure binds to RA response elements (RAREs), with modulation of the transcription regulators of several genes and the production of specific target peptides [53,56]. Accordingly, the activation of the enzyme histone acetyl transferase (HAT) is performed, occurring the chromatin decondensation over the promoter region of the target gene. Afterwards, the transcription is activated [57].

**Figure 2:**
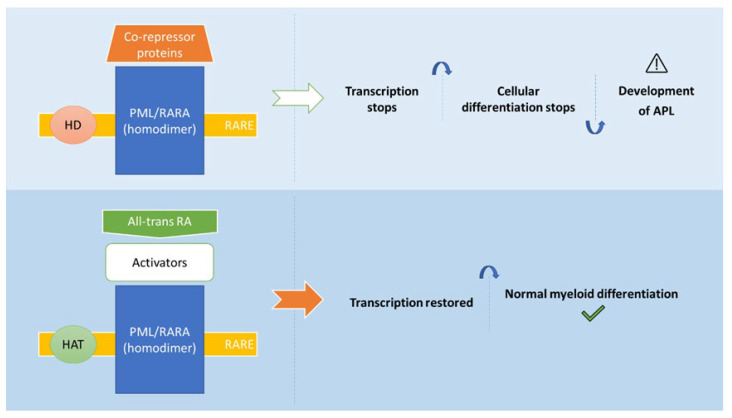
Mechanisms of action of all-trans RA in the treatment of APL (adapted from [10]). Abbreviations: All-trans RA: all-trans retinoic acid; HAT: histone acetyl transferase; HD: Histone deacetylase; PML/RARA: fusion protein (oncoprotein); RARE: RA response elements.

In APL, the PML/RARA fusion protein maintains the ability to bind to RAR and RXR, forming homodimers and heterodimers, respectively. Moreover, PML/RARA can also bind to RAREs and thus mediate the repression of RARα target genes (Figure 2). Herein, the co-repressor complex which is composed by co-repressor proteins and histone deacetylases is recruited. Subsequently, the condensation of chromatin and the gene silencing is triggered. Histone methyltransferases and DNA methyltransferases can be also recruited by the PML/RARA fusion protein, which leads to transcription prevention. Moreover, this oncoprotein has the further ability to recruit co-repressor proteins from both PML and RARα domains, occurring the formation of a stable complex with co-repressor complex. The physiological RA concentration is not enough to dissociate this complex, whereby the co-repressor complex acts on blocking the myeloid maturation at the promyelocyte stage [3,58,59,60,61,62].

## 5. Encapsulation Technology as an Ally in the Treatment of APL

Encapsulation arises as an emerging technology capable of promoting the innovation and the development of new products of industrial interest. The incorporation of bioactive compounds into carrier-based systems at a nano- and micro-scale has been widely described in the food [63,64] and pharmaceutical [65,66] sectors, providing scientific, technological and commercial opportunities to these industries. Most of these compounds are very sensitive and unstable under specific conditions (light, heat and oxygen, among others), as well as during processing and storage [67]. Accordingly, the direct integration of food- and pharmaceutical-grade bioactive compounds and ingredients to produce functional foods, medical foods and excipient foods can be a challenge for the industry [68,69,70]. This challenge increases with the highly lipophilic properties and low and/or variable bioaccessibility and bioavailability of the bioactive compounds intended to be used in products for oral ingestion [71,72,73]. The development of nano- and microstructures capable of protecting the bioactive compounds against the adverse outside surrounding environment, to promote its dispersion, to increase the solubility and to beneficiate the controlled release and absorption of lipophilic molecules at desired locations within the gastrointestinal tract is of major importance to increase the bioavailability and ultimately to improve the efficacy of target bioactive compounds in the human body [73,74,75,76]. Addressing these formulations for the addition of bioactive compounds into food and pharmaceutical products may promote the preservation, safety, quality and ability of the final product to fulfil the purpose for which it was designed.

**Figure 3:**
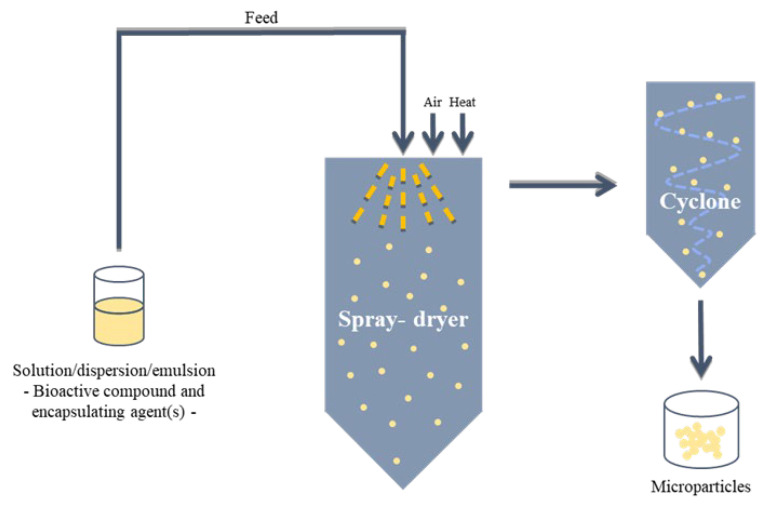
Schematic representation of spray-drying process.

Most of the carrier-based systems proposed for encapsulation of nutraceuticals and drugs include particles and capsules, nanostructured lipid carriers (NLC), solid lipid nanoparticles (SLN), hydrogels, liposomes, emulsions and self-emulsifying drug delivery systems (SEDDS) [77,78]. In some studies, the combination of encapsulating methods is considered to obtain a structure with specific characteristics according to its final application. Particles and capsules can be produced by several materials (e.g. biopolymers and synthetic polymers) using different encapsulation techniques, namely spray-drying technology. Microencapsulation by spray-drying is one of the oldest encapsulation methods [79] and is recognized to be simple and economically advantageous. Spray-drying is based on the atomization of a liquid system (a solution, dispersion or emulsion containing the bioactive compound(s) to be encapsulated and the encapsulating agent(s)) with the formation of a dry powder (Figure 3) [80]. This process is very fast (a few seconds), whereby the obtained nano- and/or microparticles are suitable for the encapsulation of heat-sensitive bioactive compounds [81]. Accordingly, spray-drying is widely used in the food industry and a common method applied in the pharmaceutical industry [82,83].

The incorporation of RA into a carrier-based system has been widely explored to improve the medical action of this retinoid in the treatment of acne, photoaging, psoriatic, several malignances (e.g. glioblastoma, lung, gastric, ovarian, prostate, stem cells and melanoma) and other diseases [84,85,86,87]. This strategy aims to provide an effective protection and stability to RA. In general, retinoids are inadequately soluble in an aqueous environment and can be damaged due to heat, the presence of oxidants and light [84]. In particular, RA is highly sensitive to oxidizing compounds, air and UV light. Therefore, a sustained RA delivery can also be achieved and at the desired place [84]. At last, undesirable side-effects related to RA administration might be mitigated [88]. The development of a bio-friendly carrier-based system for RA encapsulation emerges as an alternative approach for oral administration of this bioactive compound in the APL treatment. The ultimate aim is to increase the efficiency of RA as pharmaceutical agent. The current approach used for the RA administration and delivery to patients is an undeniable clinical challenge [84]. Herein, continuous repetitive RA oral administration (Vesanoid®) leads to a marked decline of RA half-life in blood and consequent decrease in RA concentration in the plasma [89]. This behavior can be explained by three hypothesis: (1) biliary excretion that is involved in the elimination of at least 60% of RA, whereby an unreal malabsorption or a nonspecific binding to the intestinal proteins compromises RA oral bioavailability; (2) RARα mutations can be recorded due to the continued treatment with RA and (3) the cytochrome P450 activity that is involved in RA catabolism to regulate its cellular levels and that can be induced due to the continuous treatment with this retinoid, avoiding the maintenance of RA levels in the plasma. Despite the continued treatment, the patients become resistant to RA (relapses were observed in several patients with APL). The daily dose of Vesanoid® (trade name of all-trans RA) is 45 mg/m^2^ body surface (prescribing information). The development of an innovative strategy based on carrier-based formulations capable of providing a slow and continuous RA controlled delivery in the intestine may reduce and prevent the repetitive administration of this retinoid and consequently may reduce the activity of cytochrome P450 enzymes. This might increase the RA bioavailability in patients, which will beneficiate the efficiency and then the anticancer activity of RA. Moreover, the severe side-effects described when RA is administered (e.g. hypertriglyceridemia, abdominal pain and headache [84]) might be minimized considering encapsulation technology for the development of RA controlled-delivery systems for the treatment of APL.

Encapsulation by spray-drying is a suitable technology to produce biocompatible particles to deliver RA to treat patients with APL in a direct and efficient mode. To the best of our knowledge, the current number of studies focused on RA encapsulation for the treatment of APL is scarce. The oldest studies include the incorporation of RA into liposomes to evaluate the effect of these systems on the cellular metabolism of this bioactive compound [90,91]. Parthasarathy and Mehta [90] observed that rat liver microsomes metabolized liposomal all-trans RA to a significantly lower extent when compared to free all-trans RA. Similar results were registered in F9 embryonal teratocarcinoma cells. Accordingly, the incorporation of RA into a liposomal formulation might impact on the long-term remission in APL patients. In turn, Ozpolat et al. [91] compared the CYP26A1 expression after administration of free and liposomal all-trans RA. Liposomal all-trans RA induced lower CYP26A1 expression as well as lower metabolic activity in HepG2 and NB4 cells. Moreover, the pre-treatment of cells with free all-trans RA resulted in higher metabolic activity. The obtained results suggested that the upregulation of CYP26A1 expression and all-trans RA metabolism may be involved in the clearance of this retinoid after continuous oral administration, whereby a strategy based on liposomal encapsulation and intermittent administration of all-trans RA might improve APL treatment. All-trans RA-loaded copolymer nanoparticles based on polyethylene glycol (PEG)-poly(L-lactide) and PEG-poly(e-caprolactone) were proposed by Tiwari et al. [92] to compare the differentiation induction potential of HL-60 cells with free all-trans RA. The obtained nanoparticles evidenced a moderate colloidal stability and an encapsulation efficiency of around 30%. In vitro release studies showed a RA release of 71% and 84% over two weeks from nanoparticles based on polyethylene glycol (PEG)-poly(L-lactide) and PEG-poly(e-caprolactone), respectively. Moreover, a pseudo-zero order release was evidenced. The encapsulation procedure enabled the increase in the all-trans RA photostability and showed comparable results to free all-trans RA in inducing HL-60 respiratory burst. Silva et al. [93] investigated cholesteryl butyrate SLN for the assessment of cell viability and distribution of cell cycle phases for HL-60, Jurkat and THP-1 cell lines. An increased inhibition of cell viability by all-trans RA-loaded SLN was observed when compared to free all-trans RA. In addition, an increase in sub-diploid DNA content in the cell cycle was observed for the encapsulated all-trans RA.

Until 2018, a single study described RA encapsulation using the spray-drying technology. Zuccari et al. [94] proposed a formulation based on modified polyvinyl alcohol (PVA) polymeric micelles for parenteral administration. Controlled release studies evidenced a very slow release of RA in PBS, whereby a fundamental requirement was fulfilled regarding the stability of the carrier-based delivery systems towards drug release for at least 24 h. Moreover, the cytotoxicity of encapsulated all-trans RA was higher against neuroblastoma cell lines when compared to free all-trans RA. In the most recent years, several studies were carried for the development and optimization of RA controlled-delivery systems, based on microparticle formulations obtained by spray-drying, to be used in the treatment of APL. An approach based on different types of systems—solutions and emulsions—composed by biopolymers and synthetic polymers as encapsulating agents (individually used and as a blend) has been presented for the controlled release of RA in the intestine (oral administration). Gonçalves et al. [95] proposed the incorporation of RA into microparticles individually composed by arabic gum, modified chitosan and alginic acid sodium. Herein, a RA solution prepared in ethanol was mixed with each biopolymer solution prepared in ultra-pure water and the final solutions were fed to the spray-dryer. The obtained microparticles evidenced a spherical form and a variable morphology according to the biopolymers used as encapsulating agents. Modified chitosan, alginic acid sodium and arabic gum microparticles exhibited a smooth, slightly rough and rough surface, respectively. Regarding the RA controlled-release studies, the slower release was achieved from alginic acid sodium-based microparticles (>7 h) in octanol, whereby this encapsulating agent was shown to be the most promising for RA controlled release. In turn, a faster release was observed from modified chitosan-based microparticles (99 min). However, for these formulations a high loss of RA was evidenced over time (48% and 83% from modified chitosan- and alginic acid sodium-based microparticles, respectively). Moreover, arabic gum-based microparticles lost the total amount of RA encapsulated. Gonçalves et al. [96] proposed improved formulations wherein oil-in-water emulsions composed by RA (dissolved in coconut oil), a biopolymer (alginic acid sodium, modified chitosan, arabic gum, gelatin and xanthan gum) and the surfactant Tween 80 were spray-dried to produce RA-loaded–biopolymer-based microparticles. The inlet temperature was optimized for the modified chitosan-based microparticles and the highest RA encapsulation was observed at 130 °C (65 ± 6%). Among all the microparticles that were produced, the highest RA encapsulation efficiency was obtained for modified chitosan-based and, in second place, for alginic acid sodium-based microparticles (62 ± 5%). These microparticles showed a surface with several concavities, while arabic gum-based microparticles exhibited a smoother surface. In turn, gelatin- and xanthan-based microparticles evidenced a rough surface. Regarding controlled release studies, xanthan gum and arabic gum-based microparticles resulted in a faster release of RA, while the alginic acid sodium-based microparticles again enabled the registration of the slower release of this retinoid (almost 8 h). The oil-in-water emulsions proposed by Gonçalves et al. [97] have an overall similar composition to the emulsions proposed by Gonçalves et al. [96], but contain binary and ternary blends of the biopolymers that individually enabled the production of RA-loaded microparticles with the most advantageous characteristics (xanthan gum + alginic acid sodium (50%–50%), modified chitosan + alginic acid sodium (50%–50%), modified chitosan + xanthan gum (50%–50%) and modified chitosan + xanthan gum + alginic acid sodium (33.3%–33.3%–33.3%). A simplex centroid experimental design was used to evaluate the encapsulation efficiency, loading capacity and release times of RA-loaded microparticles regarding the blends of biopolymers used as encapsulating agents. The xanthan gum + alginic acid sodium blend enabled the production of RA-loaded microparticles with the highest encapsulation efficiency (76 ± 4%). The microparticles produced evidenced a combination of characteristics typically exhibited by the microparticles individually composed by the biopolymers. Most of the microparticles composed by alginic acid sodium also enabled the RA release to be prolonged for a longer time (almost 7 h). Exceptionally, microparticles composed by the modified chitosan + alginic acid sodium blend resulted in a complete release of RA after 100 min. Gonçalves et al. [98] proposed two synthetic polymers—ethyl cellulose (EC) and polyethylene glycol (PEG)—for the development of carrier-based systems for RA encapsulation using spray-drying technology. RA was mixed in EC and EC + PEG solutions (both dissolved in absolute ethanol). These formulations enabled a significant increase in the amount of RA added to the system fed to the spray-dryer when compared to the biopolymer-based systems previously proposed [95,96,97]. The encapsulation efficiency therein obtained was around 100%, which meant that these carrier-based delivery systems became more suitable for the treatment of APL when compared to the biopolymer-based microparticles previously presented, due to the increased amount of RA encapsulated in the microparticles. The EC- and EC + PEG- based microparticles showed an irregular form and rough surface. Controlled release studies evidenced that the RA release from these synthetic polymer-based microparticles was very fast (40 min). Complementary studies were further performed to investigate the in vitro bioaccessibility and intestinal transport of RA encapsulated into EC- and EC + PEG-based microparticles. The aim was to evaluate these microparticles regarding their behavior in the gastrointestinal tract and then evaluate the EC- and EC + PEG-based microparticles for RA release and delivery in the intestine. RA bioaccessibility after in vitro static digestion (INFOGEST procedure) of RA-loaded synthetic polymer-based microparticles, with and without co-ingestion of a reference diet (Western diet) was significantly affected at intestinal level by the type of microparticles and the presence of meal. The digestion of EC- and EC + PEG-based microparticles without diet significantly increased the RA bioaccessibility from 24 ± 6 to 84 ± 1% and 25 ± 5 to 54 ± 4%, respectively. Moreover, comparing these both type of microparticles, the bioaccessibility of this retinoid was significantly higher for EC-based microparticles digested without diet. At last, co-ingestion of EC- and EC + PEG-based microparticles with diet enabled a similar RA bioaccessibility among both formulations. Regarding intestinal transport, it was observed that the amount of RA that reached the basolateral compartment was significantly influenced by the samples used in the experiments. The best results were obtained from blank digesta spiked with RA. In the most recent study, Gonçalves et al. [99] proposed the co-encapsulation of RA with curcumin and/or resveratrol. The combination of all-trans RA and arsenic trioxide is recognized as the most successful strategy for the therapy of APL patients with low and intermediate risk, as previously described (Section 3), whereby this approach is included in the front-line therapy. However, several studies have brought knowledge about new retinoids and the possibility to combine different molecules with all-trans RA or arsenic trioxide. The aim is to trigger different mechanisms of action and thus create a synergistic effect on growth control and apoptosis of malignant cells in APL [62]. Some of these molecules include curcumin and resveratrol. Curcumin is recognized for its anticancer activity, among other pharmacological functions. In particular, in APL treatment curcumin acts by inhibiting and arresting the proliferation of leukemic cells due to the stabilization of the misfolded nuclear receptor co-repressor (N CoR) protein. The accumulation of the misfolded N-CoR in the cytosol (promoted by PML/RARα) induces stress in the endoplasmic reticulum and the activation of the unfolded protein response with cytoprotective or cytotoxic action. Curcumin inhibits some proteases and the proteasomes that degrade the misfolded N-CoR, increasing stress in the endoplasmic reticulum and consequently sensitizing APL cells to apoptosis [13]. Some studies highlight that high concentrations of curcumin may trigger the apoptosis of NB4 and the combination of all-trans RA and curcumin may increase the differentiation of these cells [13,100,101,102]. Resveratrol has also received great attention due to its health benefits, namely in cancer prevention due to the induction of apoptosis and cell cycle arrest [103,104]. Resveratrol acts against APL by preventing proliferation and inducing apoptosis of cells, which can be related to the ability of this molecule to upregulate the PTEN expression and to inhibit the PI3K/AKT pathway activity [104,105]. The activity of resveratrol in the treatment of APL can be also benefit arsenic trioxide. Their combination and co-administration significantly improve the arsenic trioxide action in NB4 cells. Resveratrol can also decrease the cardiotoxicity and hepatotoxicity due to the administration of arsenic trioxide. In the co-encapsulation study performed by Gonçalves et al, microparticles loaded with RA, RA + curcumin, RA + resveratrol and RA + curcumin + resveratrol were produced. Moreover, two different microparticles’ formulations were considered as co-encapsulation systems: (i) alginic acid sodium-based microparticles (obtained from oil-in-water emulsion), due to the relatively high encapsulation efficiency and slow controlled release profiles of RA; and (ii) EC + PEG-based microparticles, due to the in vitro digestion results and the ability to encapsulate RA in an amount closer to the current daily dosage administered (this analysis is based on the previous studies presented here). Encapsulation efficiency was significantly influenced by the encapsulating agent(s) used in the microparticles’ formulations, whereby the biopolymer-based microparticles and synthetic polymers-based microparticles enabled an encapsulation efficiency for all the bioactive compounds that varied between 26 ± 3-34 ± 1% and 91 ± 6-97 ± 8%, respectively. The bioactive compounds that were co-encapsulated showed a similar release profile and it prolonged between 48 min and more than 6 h from alginic acid sodium-based microparticles and stabilized between 60 and 80 min from EC + PEG-based microparticles.

## 6. Conclusions

APL is described as a rare disease. It was first described as a potentially deadly disease, but the incorporation of chemotherapy increased complete remission rate from 13% to 55% due to the inhibition of the proliferation of APL cells. The implementation of all-trans RA in the treatment of APL markedly increased the survival rates to around 80–90%, being involved in the differentiation of leukemic promyelocytes. The most interesting option for front-line therapy of APL patients with low and intermediate risk involves the combination of arsenic trioxide and all-trans RA, with observation of better results regarding event-free survival and overall survival and a significantly lower incidence of relapse. The PML/RARA fusion protein in APL acts as a co-repressor of RARα target genes, with requirement of an increased amount of all-trans RA than physiologically observed to trigger the leukemic promyelocytes’ differentiation. The development of carrier-based delivery systems capable of providing a continuous and slow release of all-trans RA in the intestine has been investigated to increase the bioavailability of this retinoid in the human body and thus beneficiate the APL treatment. In the last few years, several studies based on all-trans RA encapsulation using spray-drying technology were reported, wherein different formulations were proposed for oral administration. A preliminary approach regarding the effect of RA, curcumin and/or resveratrol co-encapsulation in the co-release of these bioactive compounds was understood. However, the total number of studies about all-trans RA encapsulation addressed for the treatment of APL is currently still very low. Accordingly, it is of great importance that more in vitro and then in vivo protocols will be developed in this promising research field.

## Data Availability

Data Sharing Not Applicable.
